# Psychometric Properties of the Improved Report of Oslo Trauma Research Centre Questionnaires on Overuse Injuries (OSTRC-O2) and Health Problems (OSTRC-H2)

**DOI:** 10.3390/medicina61050935

**Published:** 2025-05-21

**Authors:** Giulio Leonardi, Giovanni Galeoto, Filippo Maselli, Roberto Napoli, Simone Favaretto, Martina Tomassini, Giuseppe Plebani, Lorenzo Carraro, Domenico Angilecchia

**Affiliations:** 1 University of Siena, 53100 Siena, Italy; giulioleonardi2@gmail.com (G.L.); plebani@unisi.it (G.P.); lorenzocarraro14@gmail.com (L.C.); 2 Department of Human Neurosciences, Sapienza University of Rome, 00185 Rome, Italy; masellifilippo76@gmail.com (F.M.); roberto.napoli@unisi.it (R.N.); simone.favaretto@icloud.com (S.F.); tomassini.1901777@studenti.uniroma1.it (M.T.); 3 IRCCS Neuromed, 86077 Pozzilli, Italy; 4 Sovrintendenza Sanitaria Regionale Puglia INAIL, 70126 Bari, Italy; 5 Department of Rehabilitation ASL-BA, 70123 Bari, Italy; angilecchia@gmail.com

**Keywords:** overuse injuries, sport injuries, questionnaire validation, psychometric analysis, OSTRC-O2, OSTRC-H2, Italian version

## Abstract

*Background and Objectives:* The Oslo Sports Trauma Research Centre Overuse Injury Questionnaire (OSTRC-O2) and the Oslo Sports Trauma Research Centre Questionnaire on Health Problems (OSTRC-H2) scales are designed to objectively monitor various overuse or acute injuries of professional and non-professional athletes in association with other physical problems that relate to health spheres. The aim of this study was to validate these questionnaires in a population of professional and amateur Italian athletes and to analyze their psychometric properties, in order to verify that both scales have equivalent properties in different linguistic and cultural contexts. *Materials and Methods:* The Italian versions of the OSTRC-O2 (OSTRC-O2-IT) and the OSTRC-H2 (OSTRC-H2-IT) were administered to 102 professional and non-professional athletes over a period of 3 months, once a week, for each rating scale. The inclusion criteria were: age over 18 years, practicing a sports activity for at least 1 year and having had at least one injury. The internal consistency and reliability of both scales and their correlations with pain and quality of life scales have been analyzed. *Results:* The psychometric properties of the scales turned out to be very high and statistically significant for both scales proposed to the athletes. A Cronbach’s Alpha of 0.946 and an ICC between 0.705 and 0.746 confirmed the good reliability of the questionnaires. *Conclusions:* The Italian versions of the OSTRC-O2-IT and OSTRC-H2-IT assessment scales are reliable and valid tools for the monitoring of overload injuries. This study shows that they are easy to understand for the Italian sports population and may be of help to the scientific community to increase precautionary control and prevention measures for overuse injuries in professional and amateur athletes, favoring a safer return to the field.

## 1. Introduction

In recent decades, new sports have been created due to the ever-increasing need for movement and recreation by the world population for the prevention of obesity or other comorbidities that may be linked to a sedentary lifestyle [1]. Over these years, the level of physical activity performed during the day has therefore increased significantly, in order to improve individuals’ quality of life and work productivity. However, this has also led to an increase in overuse injuries, that is, injuries caused not by a well-defined mechanic, but instead characterized by a continuous and assiduous repetition of motor patterns functioning as part of a sport-specific gesture [2]. This type of injury is often underestimated, due to the scarcity in the scientific literature of specific supervision methods for injuries that are defined as overuse injuries in various sports [3].

It is therefore of fundamental importance to identify a method for the control and monitoring of athletes to understand whether, by establishing specific protocols, it is possible to prevent or at least reduce the incidence of overuse injuries and promote greater awareness in the sports field. By implementing these protocols, it would be possible not only to improve injury prevention, but also to optimize athletic performance and the longevity of the individual’s sports career. In fact, the high incidence of overload injuries in athletes reduces sports performance and increases recovery time and the cost of health and social spending [4]. Injuries related to overload are often not considered adequately and, above all, are not studied enough to understand the impact they can have on the future of the sports season of the individual athlete and, certainly, on that of the team. Just like overt injuries of musculoskeletal origin, the various possible physical complications of a broader spectrum cannot be overlooked, especially as they often evolve into much more serious conditions, forcing athletes to abstain completely from training activities and competitions. For these reasons, the two questionnaires analyzed, in order to be effective and reliable, must be filled out weekly for a specific period of three months. They must contain specific questions regarding the individual’s ability to participate in training and sports events, in association with the presence of known and unknown injuries or physical ailments.

**Figure 1:**
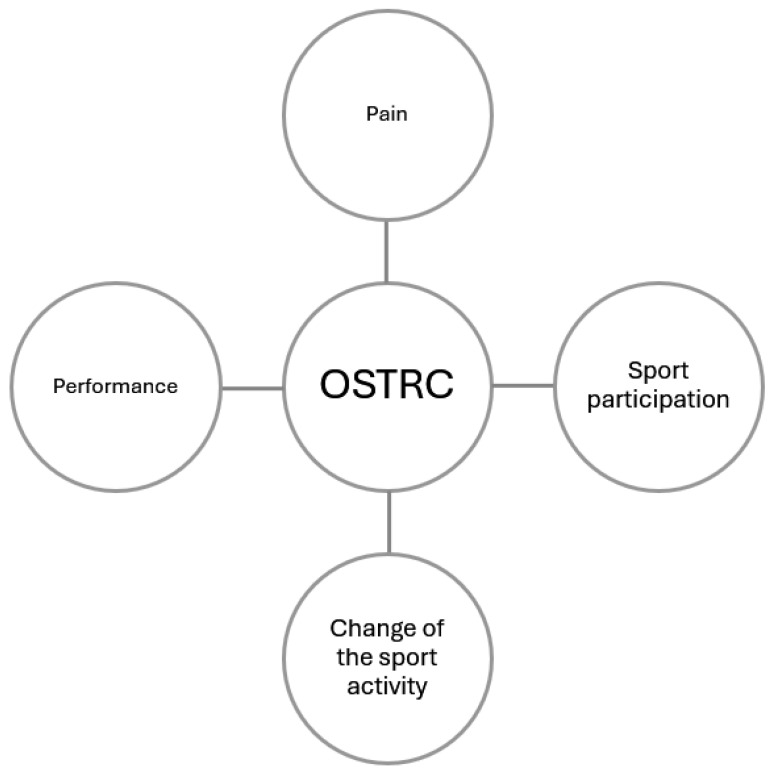
Domains of the Oslo Sports Trauma Research Center Questionnaire.

The Oslo Sports Trauma Research Overuse Center (OSTRC) Injury Questionnaire was developed and validated for the first time in 2013 by Clarsen et al. [5] to evaluate and monitor musculoskeletal overuse injuries in athletes (Figure 1). It was than translated and validated in Norwegian athletes [5], German Paralympic athletes [6], Spanish adult athletes [7], Thai-speaking athletes [8] and in the following languages: Danish [9], Swedish [10], Brazilian [11], Japanese [12] and Italian [13].

In recent years, two other versions of the questionnaire have been developed: the Oslo Sport Trauma Research Center component specific to overuse (OSTRC-O2) and the Oslo Sport Trauma Research Center component specific to general health (OSTRC-H2). These two versions have been translated and validated in Norwegian [14], Japanese [15] and Spanish [16]. Only the OSTRC specific to general health problems (OSTRC-H2) has been translated in Slovenian [17] and French [18].

The OSTRC-O2 and OSTRC-H2 questionnaires were translated and culturally adapted also in Italian, and an analysis of reliability and internal consistency was carried out [13].

Validation of a rating scale in multiple languages is crucial to ensure that its use is appropriate and that results are comparable across different populations. Validation in multiple languages makes it possible to ensure that the scale measures the same psychological constructs consistently and accurately, despite linguistic and cultural differences. Translation and cultural adaptation of the questionnaire items, in fact, are essential to avoid bias and ensure that the questions are understandable and relevant to each cultural group. An accurate adaptation process thus helps to ensure that the instrument remains useful and effective, contributing to the generalizability of the results globally [19].

Even though an Italian validated version of this questionnaire still exists, it is not validated for Italian professional and non-professional athletes [13]. For this reason, the aim of this study is to validate the OSTRC-O2 and OSTRC-H2 questionnaires for Italian athletes, in order to increase the control of overuse injuries and the knowledge of the actual prevalence and incidence of such injuries among athletes in Italy.

Moreover, in this study, differently from the first Italian validation, the following psychometric properties have been analyzed: internal consistency, reliability, construct validity and cross-cultural validity.

## 2. Materials and Methods

**Figure 2:**
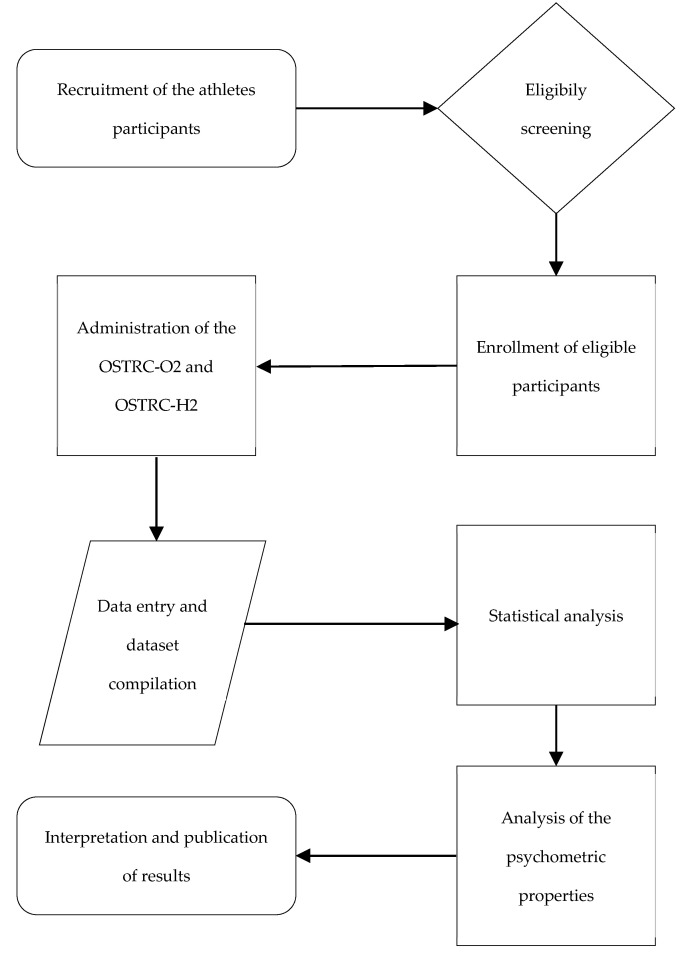
Experimental workflow of the study protocol.

The present study was a cross-sectional validation study conducted by a research group of “Sapienza” University of Rome RES—Riabilitazione Evidenze e Sviluppo, which has been involved in various studies on rehabilitation [20]. The methods used are illustrated in Figure 2.

**Inclusion:** Age over 18; practicing sport at a competitive or non-competitive level for at least one year; to have suffered of at least one injury during sport; 102 patients: 85% male; SPSS Software 29 [18].

### 2.1. Intruments

The Research Center Overuse Questionnaires Injuries (OSTRC-O2) and the Oslo Sport Trauma Research Center Health Problems (OSTRC-H2) questionnaires were first conceived and introduced in 2013 by Clarsen et al. [5].

Currently, after necessary modifications and adaptations, they consist of 4 questions each, with multiple-choice answers and, depending on the level of restriction perceived in that specific period by the athlete, only one of the 4 available answers is indicated. The timeframes to which each question refer were initially, in a general way, “in the last week”, before being adapted and specified in the modified version in 2020 with the terminology “in the last 7 days” [14]. Each questionnaire has a severity score that ranges from 0, which indicates optimal performance or health status, to 100, which refers to the worst condition [14].

Together with the two questionnaires of the OSTRC-H2 and OSTRC-O2, the Numeric Pain Rating Scale (NPRS), a subjective pain rating scale [21], and another scale to verify mental health in association with the injury to which the athlete refers, namely the SF-12 Health Survey (SF-12) [22], were also administered. The Numeric Pain Rating Scale (NPRS) is an 11-point numeric pain rating scale that allows the individual to quantify his pain from a minimum score of 0 (no pain) to 10 (the worst pain ever felt) [21].

The SF-12 is a multipurpose and generic health questionnaire consisting of 12 items, which was developed to be a shortened version of the SF-36 for assessing the quality of life of individuals. The characteristics of this scale were analyzed for the first time in the Italian population in 2007 by Kodraliu et al. Scores above 50 indicate a better-than-average health-related quality of life, while scores below 50 suggest below-average health.

### 2.2. Participants

This study was conducted in the period between May and October 2024 and all participants were recruited from various sports teams in the cities of Siena, Perugia and Treviso. All procedures followed were in accordance with the Helsinki Declaration of 1975, as revised in 2008. This study received ethical approval from the Ethics Committee of the University of Siena (CAREUS, protocol 26/2024). A written informed consent form was obtained from all the participants before being included in the study.

According to the literature, recommendations for sample size differ considerably, with suggested ratios ranging from 2 to 20 subjects per item, as reported in a recent [23] systematic review on sample sizes used in scale validation. The review found an average subject-to-item ratio of 28, with values ranging from a minimum of 1 to a maximum of 527. A minimum sample size of 80 subjects was considered adequate by the authors of this study.

The inclusion criteria were:

- Age over 18 years
- Practicing sport at a competitive or non-competitive level for at least one year
- To have suffered of at least one injury during sport

All athletes completed each of the questionnaires once a week for a period of 3 months, based on their physical and general health conditions at that specific time.

### 2.3. Internal Consistency and Reliability

An evaluation of internal consistency was carried out using Cronbach’s Alpha (α), which allowed us to make a precise calculation of the mean, standard deviation and all socio-demographic variables [7]. Cronbach’s Alpha (α) summarizes the internal correlation of the scale items and has a value that can range from 0 to 1: a value higher than 0.70 is considered acceptable [24].

To assess reliability, the OSTRC-O2 and the OSTRC-H2 were administered twice to the athletes by the same professionals. To measure the test–retest reliability, the Intraclass Correlation Coefficient (ICC) was calculated: if it has a value of ≥0.70, it is considered optimal for establishing the degree to which repeated measurements are free from measurement error, so the scale was considered stable at test–retest with an ICC greater than 0.70.

### 2.4. Construct Validity

Construct validity refers to the degree to which a test measures what it claims to be measuring, so it tests whether constructs that should be correlated are, in fact, correlated [25].

The following ranges were used to interpret the results: 0 indicates no linear relationship; +1/−1 indicates a perfect linear positive/negative relationship; ρ > 0.70 = strong correlation, 0.50 < ρ < 0.70 = moderate correlation and ρ < 0.50 = weak correlation. The significance level was set as a *p*-value less than or equal to 0.05.

To assess the construct validity, the SF-12 and the NPRS were used as comparison scales and completed together with the scales used during the first administration.

### 2.5. Cross-Cultural Validity

Cross-cultural validity is the degree to which the performance of items on a translated or culturally adapted PRO instrument are an adequate reflection of the performance of the items in the original version of the PRO instrument [26]. Cross-cultural validity refers to the degree to which evidence and theory support the interpretations and uses of test scores for different cultural groups and comparisons across groups. For the statistical analysis of the cross-cultural validity, the Student’s *t*-test for groups with two variables and the ANOVA test for groups with more than two variables were used.

### 2.6. Statistical Analysis

All the statistical analyses were carried out with the Statistical Package of Social Sciences (SPSS) 18.0. The reliability and validity of the Italian culturally adapted GSDS was assessed following the Consensus-Based Standards for the Selection of Health Status Measurement Instruments (COSMIN) checklist [27].

Statistical significance is calculated starting from a *p* value < 0.01.

## 3. Results

### 3.1. Participants

For the following study, 102 athletes of various sports, ages, sexes and with different types of pain were recruited (Table 1). There are different types of pain, which can be evoked by traumas, surgical operations and cumulative micro-traumas. Pain is called acute if present for less than 3 months, while if present for more than 3 months, it is called persistent [28].

### 3.2. Internal Consistency

The result of the Cronbach’s Alpha value, which indicates the internal consistency of the scale, obtained a value of 0.946, which is excellent and statistically significant for both subscales (OSTRC-O2; OSTRC-H2). The analysis of the Alpha deleted value showed statistically significant data; therefore, all the items of the subscales contribute to evaluating the construct of the questionnaire (Table 2).

Statistical significance is calculated starting from a *p* value < 0.01.

### 3.3. Reliability

The intraclass correlation coefficient (ICC) was used to evaluate the test–retest reliability, carried out one week later. The results were excellent for all the items of the scale and also for the total scale (Table 3), with an ICC value higher than 0.70. The period of time between the test and the retest was one week for each of the two questionnaires.

Statistical significance is calculated starting from a *p* value < 0.01.

### 3.4. Construct Validity

The construct validity of the OSTRC-O2 and OSTRC-H2 scales was calculated using the Pearson coefficient, comparing the scales with the SF-12 and the NPRS separately. Both scales obtained statistically significant values of *p* < 0.01, with low Pearson correlations (Table 4). As shown in Table 4, the value of the Pearson coefficient evaluating the correlation between the OSTRC and the SF-12 Physical Health is −0.3, meaning there is a weak correlation; so, the more the patient reports musculoskeletal problems, the lower his quality of life related to his physical health is. The value of the Pearson coefficient evaluating the correlation between the OSTRC and the SF-12 Mental Health is −0.05, so there is no correlation and this result is not statistically significant; this lack of statistical significance could result from a too-small sample or high variability in the data. There is a low correlation between the NPRS scale and OSTRC, with a Pearson value of 0.3.

### 3.5. Cross-Cultural Validity

For the cross-cultural analysis, all the characteristics of the population were used. The variables were grouped by sex, age and hours of physical activity, because these are the key demographic and behavioral variables that can influence the perception of pain, injuries and health conditions, and therefore the responses to the OSTRC questionnaires. Table 5 shows the data demonstrating that there are no statistically significant differences in the populations investigated (*p* value > 0.05).

## 4. Discussion

The aim of this study was to validate the Italian versions of the OSTRC-O2 and OSTRC-H2 scales, in order to enrich the Italian scientific literature on the prevention and, above all, on the monitoring of overload injuries in professional and amateur athletes.

The OSTRC-H2 and OSTRC-O2 questionnaire versions have been validated in different populations of athletes, offering important insights into their psychometric properties [7,8,13,14,15,16,17]. This study examined internal consistency, reliability and construct validity in a sample of 102 professional athletes with an average age of 26.44 years.

### 4.1. Internal Consistency

Internal consistency was measured through Cronbach’s Alpha, which recorded a value of 0.946, indicative of excellent internal consistency. This finding is in-line with other validations; for example, the Spanish version of the OSTRC in handball players obtained a total Cronbach’s Alpha of 0.954 [9], while the Italian validation reported values of 0.93 for the OSTRC-H2 and 0.90 for the OSTRC-O2 [15]. The French validation of the OSTRC-H2 showed even higher values of 0.97 for the overall sample and 0.94 for the subgroup with health problems [20]. Therefore, our results are consistent with previous studies that demonstrate a high reliability of the OSTRC questionnaire in different athlete populations.

Nonetheless, in a Spanish population of young athletes, the values were slightly lower: 0.93 for the OSTRC-H2 and 0.88 for the OSTRC-O2 [18]. These slight differences in Cronbach’s Alpha values can be attributed to the characteristics of the samples examined; however, we can state that both scales have a high internal correlation for their included items.

### 4.2. Reliability

Reliability was measured by the Intraclass Correlation Index (ICC). It exceeded the value of 0.70 in this study, confirming a good level of stability. This result is consistent with the Italian validation, in which the total ICC for both scales was 0.78 [15]. In the Spanish versions, the ICC was 0.87 for the OSTRC-H2 and 0.85 for the OSTRC-O2 (9.18), values similar to those of the French validation, where the ICC was 0.85 for the general sample and 0.90 for the subgroup with health problems [20]. Variations in ICC values can be explained by specific differences in participants, such as the presence of injuries or general health.

### 4.3. Construct Validity

Regarding construct validity, this study analyzed the correlation of the scales with SF-12 and NPRS. The correlation between OSTRC and SF-12 was negative and low, with values of r = −0.3 for the OSTRC-H2 and r = −0.05 for the OSTRC-O2. The low correlation with the SF-12 indicates that the OSTRC questionnaire is highly specific for monitoring sports injuries, but cannot be used to assess the overall health of the athlete, as it does not reflect either quality of life or physical and overall well-being.

The correlation with NPRS was also low (r = 0.3), showing that the scale is poorly suited for measuring pain in athletes. This value shows that the OSTRC questionnaire is not a good indicator of the severity of pain perceived by the athlete. Therefore, the questionnaire cannot be used to assess the level of acute pain, as an athlete may have a high OSTRC score for functional limitations, but a low pain score or vice versa. In support of these claims, the French validation demonstrated the absence of significant correlations with the SFMS and VAS [20], while in the Spanish version, a significant correlation was found between the OSTRC-H2 and “total time loss” (rho = 0.61) and “partial time loss” (rho = 0.54) [18], confirming that the scale is effective in monitoring time lost due to an injury. In light of these statements, we can affirm that the scale is focused on specific measures related to sports injuries, rather than general indicators of health or pain.

### 4.4. Cross-Cultural Validity

As regards cross-cultural validity, this study does not give statistically significant differences in OSTRC scores between the populations investigated based on sex, age and hours of physical activity performed. This suggests that the tool is fair and applicable to a wide range of athletes, making it useful in heterogeneous clinical contexts. The OSTRC questionnaire can therefore also be used to compare different athletes, maintaining high reliability and consistency.

This study has strengths and weaknesses that need to be considered. Among the strengths we have the relatively large number of participants and the analysis of psychometric properties that were not studied in the previous Italian validation. However, there are some significant limitations: First, an adult-only population was recruited, so the validity of the two questionnaires in a population of Italian child or adolescent athletes is unknown. In addition, the sample analyzed includes athletes from different sports, but it would be desirable to validate the scales in specific sport populations in order to study the incidence of injuries in different disciplines. Other limitations to consider include self-assessment bias, as participants have self-assessed the presence and severity of injuries, which may affect the reliability of results. In addition, the absence of objective measurements of sports injuries could limit the accuracy of the assessment and interpretation of the answers. The duration of the study (3 months) may not be sufficient to observe long-term changes in athletes’ physical condition, especially with regard to minor or chronic injuries. Finally, the predominance of male participants, who make up 85% of the total, may limit the generalizability of the results, especially as regards gender differences.

## 5. Conclusions

Based on the results obtained through the analysis of the psychometric properties of the questionnaires, we can conclude that both scales (OSTRC-O2-IT and OSTRC-H2-IT) prove to be promising tools for monitoring musculoskeletal disorders in athletes, both professional and amateur, with particular reference to overload accidents. This study provides a solid basis for improving injury prevention strategies in Italy, thus helping to prolong athletes’ sports careers and improve their post-injury performance, favoring a safer and possibly faster return to competing. However, although the questionnaires showed good reliability and high internal consistency, the validity of the construct was weak, indicating a limited correlation with instruments that assess pain and quality of life. This suggests that the scales may not fully capture the perceived impact of the injury on the overall health of the athlete. In addition, this research has several limitations that require caution in the interpretation of results.

During this study it was noted that there are few validations of the OSTRC scale in its updated version in the OSTRC-H2 and OSTRC-O2. Therefore, it is recommended that future studies should further validate the updated versions of the questionnaires in specific sports contexts with wider and more diverse samples, including objective outcome measures, to strengthen its clinical applicability and generalizability.

## Figures and Tables

**Table 1:** Sample characteristics.

	Population N° 102
Female N (%)	15 (14.7)
Hours of activity per week N (%)	
1 (0–8 h)	13 (12.7)
2 (10–17 h)	51 (50)
3 (18–28 h)	38 (37.3)
Mean activity hours ± DS	14.67 ± 5.907
Age N (%)	
1.0 (18–22 years)	24 (23.5)
2.0 (23–27 years)	48 (47.1)
3.0 (28–32 years)	17 (16.7)
4.0 (over 33 years)	13 (12.7)
Mean age ± DS	26.44 ± 6.425
Sport N (%)	
Climbing	1 (1)
Athletics	1 (1)
Basket	17 (16.7)
Box	1 (1)
Soccer	47 (46.1)
Five-a-side soccer	4 (3.9)
Cross Fit	1 (1)
American Football	1 (1)
Kite Surf	1 (1)
Padel	4 (3.9)
Swimming ball	1 (1)
Volleyball	8 (7.8)
Speed skating	7 (6.9)
Power Lifting	3 (2.9)
Rugby	2 (2)
Tennis	3 (2.9)
Acute pain N (%)	
No acute pain	76 (74.5)
Ankle	4 (3.9)
Calf	3 (2.9)
Foot	1 (1)
Hand	1 (1)
Hip	1 (1)
Knee	5 (4.9)
Low back pain	2 (2)
Neck	1 (1)
Shoulder	1 (1)
Thigh	6 (5.9)
Trunk	1 (1)
PERSISTENT pain N (%)	
No persistent pain	19 (18.6)
Ankle	13 (12.7)
Arm	1 (1)
Calf	1 (1)
Elbow	2 (2)
Groin	7 (6.9)
Hip	5 (4.9)
Knee	28 (27.5)
Lbp	7 (6.9)
Shin	2 (2)
Shoulder	11 (10.9)
Thigh	5 (4.9)
Wrist	1 (1)

**Table 2:** Alpha Deleted Analysis.

OSTRC-O2 OSTRC-H2	Medium Scale If the Element Is Deleted	Scale Variance If the Element Is Deleted	Correlation Element—Total Corrected	Correlation Multiple Quadratic	Cronbach’s Alpha If the Element Is Deleted
Q1	35.32	634.162	0.864	0.753	0.933
Q2	34.71	570.586	0.872	0.764	0.930
Q3	34.49	583.837	0.872	0.767	0.929
Q4	35.22	590.507	0.881	0.782	0.926

**Table 3:** Test–retest reliability.

Item		Test	Retest	ICC	Confidence Interval 95%	
		Mean ± DS	Mean ± DS		Lower Limit	Upper Limit
OSTRC-O2	1	11.25 ± 7.872	12.09 ± 7.703	0.740	0.637	0.816
	2	11.87 ± 9.203	12.43 ± 8.620	0.726	0.620	0.806
	3	12.09 ± 8.907	12.41 ± 8.620	0.800	0.718	0.861
	4	11.36 ± 8.701	11.57 ± 8.411	0.746	0.646	0.821
	TOT	46.58 ± 32.229	48.50 ± 31.007	0.705	0.854	8.562
OSTRC-H2	1	11.25 ± 7.872	12.09 ± 7.703	0.740	0.637	0.816
	2	11.87 ± 9.203	12.43 ± 8.620	0.726	0.620	0.806
	3	12.09 ± 8.907	12.41 ± 8.620	0.800	0.718	0.861
	4	11.36 ± 8.701	11.57 ± 8.411	0.746	0.646	0.821
	TOT	46.58 ± 32.229	48.50 ± 31.007	0.705	0.854	8.562

OSTRC-O2: Oslo Sport Trauma Research Centre Overuse Injury Questionnaire; OSTRC-H2: Oslo Sport Trauma Research Centre Questionnaire on Health Problems; ICC: Interclass Correlation Coefficient.

**Table 4:** Construct validity.

	SF-12 PCS	SF-12 MCS	NPRS
OSTRC-O2	−0.322 **	−0.055	0.339 **
OSTRC-H2	−0.322 **	−0.055	0.339 **

** *p* < 0.01.

**Table 5:** Cross-cultural analysis.

	Mean ± DS OSTRC-O2 OSTRC-H2	Test	*p*
Female	45.90 ± 31.985	−0.513	0.609
Male	50.53 ± 34.488		
Hours of activity			
1 (0–8 h)	52.62 ± 33.306	0.262	0.770
2 (10–17 h)	45.41 ± 31.292		
3 (18–28 h)	46.08 ± 33.726		
Age			
1.0 (18–22 years)	43.50 ± 33.905	0.249	0.862
2.0 (23–27 years)	49.17 ± 31.968		
3.0 (28–32 years)	42.88 ± 32.913		
4.0 (over 33 years)	47.54 ± 32.033		

## Data Availability

The raw data supporting the conclusions of this article will be made available by the authors on request.
